# Metabolic Vulnerabilities as a Therapeutic Target in Breast Cancer

**DOI:** 10.3390/curroncol33020129

**Published:** 2026-02-23

**Authors:** Sabrina Guo, Christina L. Addison

**Affiliations:** 1 Cancer Research Program, Ottawa Hospital Research Institute, 501 Smyth Road, Ottawa, ON K1H 8L6, Canada; sguo@ohri.ca; 2 Department of Biochemistry, Microbiology and Immunology, University of Ottawa, 451 Smyth Road, Ottawa, ON K1H 8L1, Canada; 3 Department of Medicine, University of Ottawa, 451 Smyth Road, Ottawa, ON K1H 8L1, Canada

**Keywords:** breast cancer, metabolism, prognosis, tumor growth, glycolysis, pentose phosphate pathway, serine biosynthesis, glutaminolysis, fatty acids, TCA cycle

## Abstract

Metabolic reprogramming is a defining feature of breast cancer, enabling tumor cells to sustain rapid proliferation, survive under stress, and resist therapy. Key pathways including glycolysis, glutaminolysis, lipid metabolism, and one-carbon metabolism, play central roles in meeting the energetic and biosynthetic demands of malignant cells. Enhanced glycolytic flux supports ATP generation and lactate production, while glutamine metabolism fuels the tricarboxylic acid cycle and provides nitrogen for nucleotide synthesis. Lipid metabolic pathways, particularly fatty acid synthesis, contribute to membrane biogenesis and signaling, and one-carbon metabolism driven by serine and glycine supplies methyl groups for epigenetic regulation and nucleotide production. These metabolic adaptations not only promote tumor growth but also create vulnerabilities that can be exploited therapeutically. Inhibiting these pathways has shown promise in preclinical models; however, challenges such as metabolic plasticity, tumor heterogeneity, and potential toxicity in normal tissues underscore the need for biomarker-driven strategies and rational combination therapies. Herein, we describe current knowledge of the role of these pathways in breast cancer progression, highlighting the role of key enzymes in promoting breast cancer tumor cell growth and in breast cancer prognoses.

## Simple Summary

Breast cancer cells change the way they use nutrients to grow and survive, which makes their metabolism an important area for new treatments. These cells often rely on specific pathways, such as those that process sugar, amino acids, and fats, to produce energy and build essential components. Recent research shows that blocking these pathways can slow cancer growth and make existing treatments more effective. However, better understanding of these metabolic changes and their roles in different breast cancer subtypes could lead to more personalized therapies and improve outcomes for patients in the future.

## 1. Introduction

Breast cancer is the leading cause of cancer and cancer death in women, with 1.9 million new cases and 495,000 deaths globally in 2022 [1]. Despite advancements in diagnosis and therapy, mortality rates remain high, largely due to treatment resistance [2]. As a result, researchers are increasingly exploring alternative features driving cancer progression and drug resistance to identify new vulnerabilities that could be targeted for more effective breast cancer treatment. 

In recent years, altered cancer metabolism has emerged as a cancer driver and is now recognized as a ‘hallmark’ of cancer [3]. Oncogene over-expression (e.g. Myc, PI3K/Akt, KRAS, and Her2), or loss of key tumor suppressors (e.g. TP53 and BRCA1) can lead to altered metabolism that support tumor cell growth and survival [4,5,6,7,8,9]. These genetic alterations converge on key metabolic pathways, including glycolysis (affected by e.g. MYC, BRCA, KRAS), the tricarboxylic acid (TCA) cycle (BRCA, MYC), nucleotide biosynthesis (MYC, KRAS, TP53, PTEN), and lipid and fatty acid production (IDH1/2, KRAS, TP53, PI3KCA). Additionally, tumor cells shift their dependency towards non-glucose nutrients such as glutamine (e.g. MYC, KRAS, IDH1/2, TP53, PI3KCA) [10,11,12,13,14,15] or serine metabolism (MYC, KRAS, TP53) [16,17,18,19,20]. 

In breast cancer, metabolic reprogramming is associated with disease progression [21], therapeutic resistance [22], and metastatic potential [21]. Breast cancer subtypes are also associated with unique metabolic alterations with triple negative breast cancer (TNBC) exhibiting more dependance on glycolytic activity [23,24] whereas luminal tumors depend more on oxidative phosphorylation (OXPHOS) or lipid metabolism [25].

**Figure 1:**
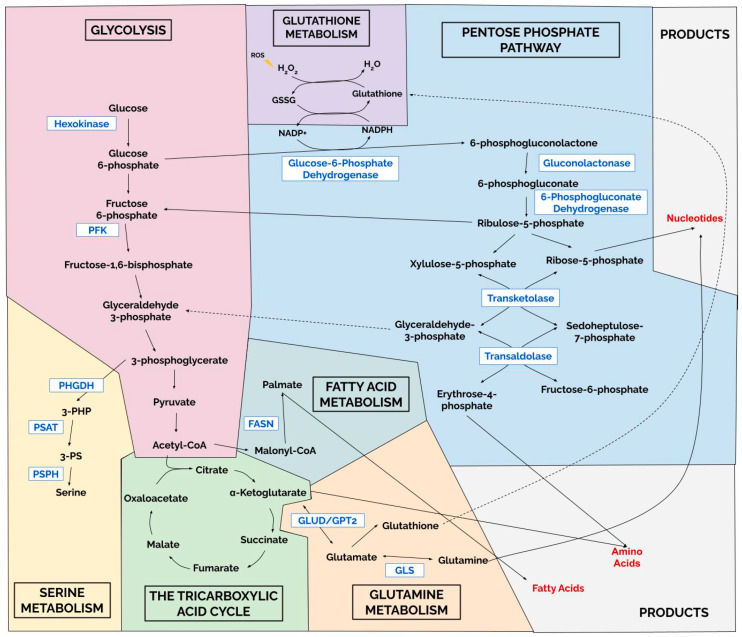
Metabolic pathways driving breast cancer growth. Breast cancers have an altered metabolism to support their growth and progression, including alterations in glycolysis, the tricarboxylic acid cycle, the pentose phosphate pathway, and serine and glutamine biosynthesis. Alterations in fatty acid and glutathione production have also been observed. Targeting key metabolic enzymes regulating these pathways holds promise for the therapeutic treatment of breast cancer. Original image created by the authors using Google Drawings (https://docs.google.com/drawings/d/1l3oCAeTKWKZzrwQXozFC4T5H2cpWQf3yhiQVIE-8Scc/edit?usp=sharing (accessed on 11 June 2025)). FASN: fatty acid synthase, GLS: glutaminase, GLUD: glutamate dehydrogenase, PFK: phosphofructokinase, PHGDH: Phosphoglycerate dehydrogenase, PSAT: phosphoserine aminotransferase, and PSPH: phosphoserine phosphatase.

Previous reviews have emphasized the role of metabolic rewiring in cancer broadly. In this review, we aim to synthesize recent findings specific to breast cancer, with a primary focus on identifying metabolic vulnerabilities that may be therapeutically exploited, while also highlighting the prognostic relevance of dysregulated metabolic pathways. We provide an updated view of pathways shown to regulate breast cancer progression (Figure 1), including glycolysis, the TCA cycle, amino acid and nucleotide biosynthesis, as well as lipid, glutamine, and serine metabolism.

## 2. Altered Metabolic Pathways Associated with Breast Cancer Progression

### 2.1. Glycolysis

Glycolysis is a key energy-generating pathway utilized by cells that converts simple sugars into pyruvate, generating two ATP molecules in the process. Pyruvate can be converted to lactate in an oxygen-independent reaction generating two additional ATP molecules, or it can enter the mitochondrial TCA cycle to generate an additional 30 ATP molecules through oxygen-dependent reactions [26]. Despite glycolysis being less energy-efficient, most breast cancers are dependent on aerobic glycolysis [27,28,29], leading to increased production of lactate compared to normal cells. Interestingly, mesenchymal tumor cells exhibit even greater dependence on glycolysis compared to epithelial tumor cells [30,31], and use locally generated ATP pools to increase cytoskeletal modeling and cell invasion [30]. Tumor cell growth in high glucose further promotes mesenchymal cell phenotypes with enhanced migratory abilities [32], supporting the notion that increased glycolysis promotes more aggressive tumor growth and metastasis. Aerobic glycolysis use is a common metabolic feature across breast cancer subtypes, with TNBC being the most reliant on this process [33,34,35], and breast cancers show significant upregulation of numerous glycolytic enzymes. We will briefly summarize the role of key glycolytic enzymes in breast cancer growth and progression

#### 2.1.1. Hexokinase 2

Hexokinase 2 (HK2), the first rate-limiting enzyme of glycolysis, is upregulated in ~40–80% of breast cancers [36,37]; however, its expression varies across molecular subtype [38]. HK2, a hypoxia-regulated gene [39,40,41], catalyzes the phosphorylation of hexose (i.e. glucose or fructose) to hexose-6-phosphate resulting in its cellular retention and glycolytic metabolism. HK2 protein levels and activity are frequently elevated in breast cancer cells compared to normal mammary epithelial cells [36,37,42,43], however more recent high-throughput patient data suggest that HK2 upregulation is most prominent in Her2 positive breast cancer [38]. HK2 protein levels also correlate with histological grade, recurrence, risk of metastasis and reduced disease-free survival in breast cancer [36,44,45]. HK2 expression is also elevated in tumors with dysregulated ALK [46], MYC [47], BRCA1 loss or mutation [48], Her2 overexpression [49], PI3KCA mutation [50], or KRAS mutation [51]. 

Preclinical work supports a role for HK2 in breast cancer progression and metastasis. In transgenic breast cancer models, HK2 is required for tumor initiation and maintenance [52], and its depletion inhibited tumor growth [53,54,55]. miRNA-mediated HK2 depletion by miR-let7-b-5p [56] and miR-216b [43] also impaired breast tumor growth, whereas overexpression of miR-155 [40] or the hepatocellular carcinoma (HCC)-associated long non-coding RNA (HANR) [57] enhanced aerobic glycolysis and breast cancer tumor growth. HK2 expression is also increased in brain metastatic derivatives compared to parental breast cancer cell lines [44], contributing to metastasis through modulation of epithelial to mesenchymal transition (EMT), cell migration and cancer stem cell (CSC) phenotype [49,52,58,59,60]. Although not yet demonstrated in breast cancer, HK2 has been shown to alleviate oxidative stress, modulate pro-apoptotic proteins, and regulate autophagy [61,62,63,64,65,66,67], processes that facilitate metastatic tumor growth.

Emerging evidence suggests that HK2 and glycolytic activity contributes to tumor immune evasion. In breast cancer patient samples, a strong correlation between HK2 and PD-L1 expression was noted, with high co-expression associating with decreased immune infiltration and worse prognosis [68]. The authors also demonstrated that in high glucose, HK2 phosphorylated IκBα, thereby alleviating its suppression of NFκB leading to increased transcription of PD-L1 [68]. Interestingly, over-expression of PD-L1 in lung cancer cells increased HK2 levels, suggesting the possible existence of a positive feedback loop [69]. The authors also showed that co-culture of HK2 overexpressing tumor cells with T-cells resulted in reduced interferon (IFN)γ secretion and expression of T-cell activation markers suggesting ability of HK2 activity to suppress T-cell function. 

Given its association with promoting tumor metastases and immune suppression, inhibition of HK2 may offer therapeutic benefits for breast cancer. HK2 activity could confer resistance to tamoxifen and paclitaxel in breast cancer [70,71], while its inhibition conferred sensitivity to radiotherapy [53,72] and oncolytic virus therapy [73], supporting its potential as a therapeutic target. Towards this end, early studies tested pharmacological inhibitors targeting the glucose binding site in HK2. The glucose analogue 2-deoxyglucose inhibits HK2 activity, resulting in increased tumor cell apoptosis [74] and sensitization to chemotherapy and radiotherapy [75]. However, studies suggest it is more effective when used in combination with other metabolic modulators including hydroxychloroquine [76], mitochondrial targeting drugs [77], calcium channel blockers [78] and metformin [79,80]. Notably, combination treatment with metformin led to decreased PD-L1 expression in tumor cells [80], suggesting possible synergy with immune checkpoint inhibitors. More recent studies show that the HK2 inhibitor 3-bromopyruvate (3BrPA [81]) impaired syngeneic 4T1 breast cancer tumor growth in vivo, due in part to reduced myeloid derived suppressor cell (MDSC) tumor infiltration [55]. Treatment with 3BrPA also sensitized tumors to anti-PD-L1 treatment, which was associated with increased CD8+ T-cell and decreased MDSC infiltration. Other FDA-approved agents such as the anti-fungal medication ketoconazole have also been shown to inhibit HK2 and sensitize tumors to radiotherapy [72].

Although the agents described above are selective HK2 inhibitors, many also affect additional targets and pathways. To specifically target HK2 activity, investigators have used a virtual ligand screening approach to identify the FDA-approved drug Benserazide as a potent HK2 inhibitor [82] and demonstrated its ability to inhibit colorectal tumor growth in vivo. Zheng et al. also used an in silico screen and identified benitrobenrazide (BNBZ) as a selective HK2 inhibitor with nM potency [83]. Although not yet tested in breast cancer, BNBZ reduced glycolysis and proliferation, induced tumor cell apoptosis in vitro, and inhibited xenograft tumor growth in vivo with no detectable toxicity [83]. Despite the identification of these more selective agents, they still retain alternative target activity. To overcome this, Sang et al. [54] used a proteolysis-targeting chimera (PROTAC) approach to achieve HK2 degradation. Their lead compound effectively degraded HK2 protein, reduced glycolysis, and induced mitochondrial damage to inhibit breast cancer cell growth [54]. HK2 degradation also led to pyroptotic cell death, leading to increased anti-tumor immunity and reversal of the immunosuppressive tumor environment. Given that PROTAC agents have successfully advanced to Phase III clinical trials in cancer [84], it will be of great interest to see if PROTAC agents targeting cancer metabolism will become promising therapies. 

#### 2.1.2. Phosphofructokinases

Phosphofructokinase (PFK) enzymes, namely PFK1 and PFK2, play a rate limiting role in glycolysis converting fructose 6-phosphate to fructose-1,6-bisphosphate or fructose-2,6-bisphosphate respectively. Humans express three major PFK1 isoforms (PFKL (liver), PFKM (muscle), and PFKP (platelet)), and four PFK2 isoforms (PFKFB1-4). 

Among PFK1 isoforms, PFKP is the predominant isoform upregulated in breast cancer tumors compared with normal tissue [85] and is the only isoform associated with patient survival [86]. Elevated PFKP correlates with increased nodal metastasis and worse recurrence-free and overall survival, particularly in TNBC, which has higher PFKP expression than luminal subtypes [87,88]. PFKP upregulation is also linked to tumors with MYC over-expression [89], BRCA1 loss or mutation [88], KRAS mutation [90], or activation of the PI3K/Akt pathway, which regulates its activity [91]. PFKP promotes breast cancer progression, as RNA interference-mediated depletion of PFKP reduced tumor cell growth and invasion in vitro [92] and decreased TNBC xenograft growth in vivo [87,93]. Similarly, ubiquitin mediated degradation of PFKP via overexpression of the E3 ubiquitin ligase HRD1, reduced glycolysis, proliferation and invasion in vitro, and significantly reduced tumor growth and metastasis in vivo [94]. Use of quercetin, a flavonoid compound that inhibits PFKP and HK2 activity, reduced PFKP expression and inhibited glycolysis and proliferation in TNBC cells [87]. Although not yet tested in breast cancer, the PFKP inhibitor 2,5-Anhydro-D-glucitol-1,6-diphosphate inhibited non-small cell lung tumor cell growth in vitro [95]. Taken together, these findings suggest that targeting PFKP may effectively prevent breast cancer growth, particularly the TNBC subtype.

The PFK2 isoform PFKFB3 is transcriptionally upregulated by hypoxia and estrogen, and its promoter contains consensus binding sites for the early growth response 1 (EGR1) transcription factor [96,97]. Wild-type p53 suppresses PFKFB3 expression [98], suggesting certain TP53 mutations may lead to its upregulation. Although not demonstrated in breast cancer specifically, PFKFB3 expression can also be upregulated by EGFR [99], HER2 [100], MAPK signaling [101], MYC overexpression [102], and KRAS [103], PTEN [104], or BRCA1 [103] mutations. PFKFB3 protein stability is also regulated by the long non-coding (lnc)RNA Actin Gamma 1 Pseudogene (*AGPG*) which binds to PFKFB3 and prevents its ubiquitin-mediated protein degradation [105]. Clinically, PFKFB3 is associated with nodal invasion, distal metastasis and worse overall survival in TNBC [106,107] and Her2+ [100,107] breast cancers, whereas its prognostic role luminal breast cancer remains unclear. A recent study confirmed a positive correlation of PFKFB3 with Nottingham grade, however correlations differed between protein and RNA, with the latter showing some correlation with ipsilateral breast tumor recurrence following radiotherapy in luminal A cancers [108]. PFKFB3 promoted breast cancer tumor growth in multiple models. RNA interference-mediated depletion of PFKFB3 reduced growth of Her2+ [100] and TNBC [106] tumor cells in vitro and in vivo. In TNBC models, PFKFB3 inhibition suppressed angiogenesis and tumor growth by decreasing tumor VEGF expression [106]. Overexpression of PFKFB3 enhanced CSC properties and promoted escape from dormancy driving metastatic tumor outgrowth [107]. PFKFB3 activity has also been linked to chemoresistance [109].

Pharmacological PFKFB3 inhibition using 3-(3-pyridinyl)-1-(4-pyridinyl)-2-propen-1-one (3PO), inhibited the growth of TNBC and Her2+ tumor xenografts [100,110]. The PFKFB3 inhibitor PFK158 enhanced sensitivity of ER+ breast cancer xenografts to fulvestrant [111]. PFK158 completed Phase I clinical testing, where it was well tolerated and showed inhibitory effects on immunosuppressive immune cells [112], however no further clinical development has been reported, suggesting limited efficacy as a monotherapy. 

Another PFK2 isoform, PFKFB4 is also upregulated in breast tumors compared to normal tissue and is strongly associated with worse prognosis [113,114,115,116]. PFKFB4 expression is induced by hypoxia [115,117] and loss of p53 expression [118]. Interestingly, PFKFB4 is particularly elevated in hypoxic tumor regions, and its depletion in orthotopic TNBC models decreased metastatic burden, partly through decreased integrin β3-dependent cell invasion [115] or lactate production [119]. PFKFB4 depletion also impaired ER+ xenograft tumor growth [113], while its overexpression increased TNBC and ER+ tumor cell invasion in vitro and tumor growth in vivo [113,116]. PFKFB4 also contributes to breast cancer ‘stemness’, as the CD44ICD isoform known to enhance stem-like properties increased PFKFB4 activity [120]. The link between PDKFB4 expression and enhanced stemness was confirmed in ER+ breast cancers, and contributed to palbociclib resistance [121]. PFKFB4 activity can also promote angiogenesis, as conditioned media from tumor cells with depleted PFKFB4 had reduced ability to induce HUVEC sprouting in Matrigel assays [122]. This was attributed to enhanced lactate secretion, which led to IL-6 upregulation via NFκB, which in turn led to STAT5-mediated angiogenesis in endothelial cells. A selective PFKFB4 inhibitor, 5-(*n*-(8-methoxy-4-quinolyl)amino)pentyl nitrate (5MPN), reduced glycolysis and proliferation in numerous tumor cell lines, including MDA-MB-231, and inhibited syngeneic lung tumor growth in vivo [123]. 5MPN also inhibited the growth and metastasis of CD44ICD expressing E0771 TNBC tumors in vivo concomitant with downregulation of expression of CSC-associated genes Sox2, NANOG and Oct4 [120]. 5MPN also inhibited ER+ breast tumor xenograft growth in vivo, in part via inhibition of angiogenesis [122]. Taken together, these findings highlight the important role of phosphofructokinases in regulating key intrinsic and extrinsic mechanisms to drive breast cancer tumor growth.

#### 2.1.3. Lactate Dehydrogenases

Lactate dehydrogenases (LDH) catalyze the final step of glycolysis, converting pyruvate to lactate. As pyruvate can also enter the TCA cycle, LDH is a key determinant of whether cells preferentially utilize cellular glycolytic or mitochondrial metabolism. LDH enzymes are tetramers composed of two ubiquitously expressed subunits, LDH-A (also known as muscle or M-type) and LDH-B (also known as heart or H-type), which combine in different ratios to form 5 isoforms [124]. A sixth isoform, LDH-C, is comprised entirely of a testis specific subunit [125], but can also be aberrantly expressed by tumor cells [126]. Most clinical studies examining LDH in breast cancer have measured total circulating LDH levels rather than specific isoforms. Generally, elevated circulating LDH levels are associated with increased metastasis and worse progression-free or overall survival in breast cancer [127,128,129,130].

LDH-A expression is higher in breast tumors compared with normal breast tissue [131,132], and high LDH-A levels correlate with increased metastasis, and worse recurrence-free and overall survival [131,132,133,134]. Increased LDH-A expression is also associated with TP53 loss or mutation [131,135], MYC activity [136,137], BRCA mutation [138], and HER2 overexpression [139]. Her2 can also directly phosphorylate LDH-A enhancing its enzymatic activity [140]. Notably, tamoxifen resistant MCF7 cells have increased levels of LDH-A compared to parental tamoxifen sensitive cells [141]. Functionally LDH-A promotes breast cancer progression, as its overexpression can overcome p53-mediated inhibition of MCF7 tumor growth in vivo [135], and increase BT549 cell growth, migration and invasion in vitro, in part through upregulation of MMP2 and MMP9 [131]. Conversely, LDH-A depletion via RNA-interference inhibited breast cancer cell line proliferation, migration and invasion in vitro [131,142], and suppressed metastasis in vivo [140,142]. Pharmacological LDH-A inhibition using Oxamate [143] or FX11 [3-dihydroxy-6-methyl-7-(phenylmethyl)-4-propylnaphthalene-1-carboxylic acid] [144] also inhibited tumor growth in vitro [139,144,145,146,147] and in vivo [145,146,147,148]. Additional studies in other cancer models, suggest that LDH-A inhibition leads to reduced cancer stemness [149] and enhanced sensitivity to immune checkpoint inhibitors [150,151,152], radiotherapy [149], paclitaxel [146,153], and other metabolic inhibitors [144]. 

There are few studies that have examined the specific role of LDH-B in breast cancer. One study found LDH-B was more highly expressed in basal-like breast cancers irrespective of hormone receptor status, with higher LDH-B expression associated with worse recurrence-free survival [154]. In slight odds with this finding, high LDH-B levels were also associated with better pathological complete response (pCR) to neoadjuvant therapy [154]. In contrast, LDH-B gene expression was lower in tumors than in normal breast tissue [155]. In preclinical models, LDH-B overexpression reduced tumor cell migration and invasion without affecting proliferation in vitro, which translated into reduced lung metastatic tumor burden in vivo. Mechanistically, LDH-B overexpression shifted metabolism towards greater TCA cycle activity, reduced lactate secretion, and increased intracellular reactive oxygen species (ROS). Intriguingly, the authors demonstrated that the resulting reduced lactate production increased NK cell activation, which contributed to impaired tumor growth in vivo. Supporting an inhibitory role for LDH-B, a previous study reported reduced LDH-B expression in breast cancer cell lines compared to normal human mammary epithelial cells (HMEC), and showed that LDH-B overexpression decreased tumor cell growth by inducing mitochondrial damage and apoptosis [156]. Although additional work is needed to clarify the role of LDH-B in breast cancer, current data suggests that higher LDH-B levels may promote a tumor growth-inhibitory immune microenvironment and be associated with better prognosis. 

In breast cancer tissues, LDH-C expression shows a modest, although not statistically significant, trend towards higher levels in tumor tissue compared to normal mammary tissue [157]. However, when analyzed by subtype, LDH-C was specifically elevated in TNBC tumors. Depletion of LDH-C in TNBC cell lines led to genomic instability, increased DNA damage, and increased mitotic catastrophe, ultimately promoting increased apoptosis. Inhibition of LDH-C also sensitized cells to the DNA repair inhibitor Olaparib and the DNA damaging agent cisplatin. Bioinformatic analysis of TCGA datasets also showed that LDH-C expression positively correlates with CD4+ T-cell infiltration across breast cancer subtypes and with B-cell infiltration in Her2+ tumors [158], while a negative correlation with NK cell infiltration was observed in TNBC tumors. Mechanistically, LDH-C depletion increased IFNγ secretion, which promoted enhanced T-cell mediating killing of LDH-C-deficient breast cancer cell lines in vitro, concomitant with reduced immune checkpoint protein expression in T-cells. Therapeutic LDH-C inhibition remains limited due to a lack of isoform-specific inhibitors. However, recently use of cell-penetrating peptides (CPP) to deliver LDH-C specific siRNA molecules led to effective LDH-C depletion, inhibition of clonogenic growth, and increased sensitivity to Olaparib in vitro [159]. CPP-mediated siRNA delivery also led to a ~50% reduction in TNBC tumor growth in zebrafish xenograft models. 

Based on the evidence described above, and the established dependence of breast cancer cells on glycolysis, targeting key glycolytic enzymes remains an attractive therapeutic strategy. Inhibiting glycolysis has the potential to affect CSC phenotypes, cellular invasive properties, and immune-mediated anti-tumor activities in vivo. However, several challenges remain. Glycolysis is essential for normal cell function, particularly in tissues with high energy demands (e.g. brain and muscle cell types) raising concerns about toxicity. Inhibition of glycolysis may also lead to cellular compensation, including increased oxidative phosphorylation or glutaminolysis to promote tumor cell growth. These limitations highlight the need to identify inhibitors that selectively target tumor-enriched enzyme isoforms or explore use of metabolic inhibitors in combination therapies that limit metabolic compensation while minimizing toxicity to normal tissues.

### 2.2. TCA Cycle

The tricarboxylic acid cycle (TCA cycle, aka Krebs cycle) is a metabolic pathway that typically follows glycolysis and operates within the mitochondria to generate ATP and essential metabolites [160]. Pyruvate produced by glycolysis is transported into mitochondria and converted to acetyl-CoA and NADH by the pyruvate dehydrogenase complex (PDC). Citrate synthase (CS) then catalyzes a reaction between acetyl-CoA and oxaloacetate to generate citrate, which is subsequently converted to isocitrate, α-ketoglutarate (AKG), succinyl-CoA, succinate, fumarate, maleate, and finally oxaloacetate through a series of enzymatic reactions. Through these reactions, cellular NADH, FADH_2_, CO_2_, GTP (ATP) and crucial metabolic intermediates required for nucleotide, amino acid, and lipid biosynthesis are generated. Due to this, TCA cycle usage is frequently altered in cancer to support rapid cellular proliferation and cell survival under metabolic stress. We will highlight a few of the critical metabolites and their key regulating metabolic enzymes that have been implicated in breast cancer progression. 

#### 2.2.1. α-Ketoglutarate and 2-Hyrdroxyglutarate

α-Ketoglutarate (AKG, aka 2-oxoglutarate) is a ketoacid produced from reversible decarboxylation of isocitrate by NADP-dependent isocitrate dehydrogenases (IDH1-3). IDH1 is localized in the cytoplasm and IDH2 and 3 are localized to mitochondria [161,162]. AKG can also be generated from deamination of glutamine and glutamate via mitochondrial glutaminase (GLS) and glutamate dehydrogenase (GDH) respectively. AKG, a cofactor for numerous cellular biochemical reactions, supports cell growth and survival through generation of ATP, providing precursors for amino acid generation (e.g. glutamate, aspartate and glutamine), and alleviating oxidative stress [163]. AKG can also undergo reductive carboxylation to regenerate citrate and support lipid biosynthesis [164,165,166]. In breast cancer, AKG generally inhibits tumor growth. Treatment of breast cancer cell lines with exogenous AKG inhibits tumor cell growth and colony formation in vitro [167,168]. Moreover, inhibition of α-ketoglutarate dehydrogenase (AKGD) using the inhibitor, (S)-2-[(2,6-dichlorobenzoyl) amino] succinic acid (AA6) decreased 4T1 tumor cell invasion in vitro and lung metastasis growth in vivo [169]. 

Although cellular AKG can suppress tumor growth, mutations in IDH genes, while relatively uncommon in breast cancer, are biologically relevant. Mutant IDH enzymes generate 2-hydroxyglutarate (2HG), which acts as a competitive inhibitor against AKG-dependent enzymes, including AKG-dependent dioxygenases [170,171]. Among these are the ten-eleven translocation (TET) family of DNA hydroxylases, key tumor suppressor proteins required for demethylation of 5-methylcytosine (5mC) [172]. Although rare, IDH mutations have been reported in breast cancer [173], and the uncommon subtype solid papillary carcinoma with reverse polarity (SPCRP) which frequently harbors IDH2 mutations [174]. Interestingly, aberrant IDH activity may not require mutation, as high levels of wild-type IDH2 can also generate 2HG [175], and are associated with increased lymphovascular invasion and worse survival in breast cancer patients [176,177]. IDH2 is generally elevated in breast cancers compared to normal mammary tissue, with the highest levels observed in TNBC, and high IDH2 correlates with worse relapse-free and overall survival [178]. IDH2 depletion inhibited breast cancer cell line growth and invasion in vitro supporting its potential as a tumor driver [177,178]. IDH2 depletion also inhibited while IDH2 overexpression promoted lung metastatic breast cancer growth in vivo, with higher IDH2 expression observed in metastatic compared to primary tumors [178]. Pharmacological IDH2 inhibition by AGI-6780 (*N*-Cyclopropyl-4-(3-thienyl)-3- [[[[3-(trifluoromethyl)phenyl]amino]carbonyl]amino]-benzenesulfonamide) [179] also decreased breast cancer cell growth and colony formation in vitro, via increasing AKG and reducing ATP levels [178]. AGI-6780 treatment also impaired orthotopic MDA-MB-231 tumor growth associated with increased tumor AKG levels in vivo, while increasing AKG using the cell-permeable analogue 2-methyl-α-ketoglutarate similarly inhibited tumor growth and sensitized tumors to doxorubicin. 

Metabolomic profiling of breast cancer patient tumors showed significantly increased levels of 2HG in tumors compared to normal mammary tissues, with TNBC exhibiting higher 2HG levels than ER+ tumors [180]. Consistent with its role in inhibiting TET family enzymes, treatment of cells with exogenous 2HG reduced 5-hydroxymethylcytosine (5hmC) levels and genome-wide DNA hypermethylation. Although no mutations in IDH were found in this patient cohort, a correlation between MYC activation gene signatures and elevated levels of 2HG were observed. This association was confirmed by upregulation or suppression of MYC expression in tumor cell lines, which was associated with increased or decreased 2HG levels respectively. Other metabolic enzymes can also generate 2HG under certain conditions, including malate dehydrogenase (MDH), 3-phosphoglycerate dehydrogenase (PHGDH), and LDH-A [181]. These findings suggest that regulation of AKG and 2HG is more complex than initially appreciated, and that perhaps assessing their relative metabolic levels may be a useful prognostic marker. Regardless, supporting data suggests that strategies to decrease 2HG and increase AKG levels may suppress tumor growth, warranting further study of this metabolic axis.

#### 2.2.2. Succinate and Fumarate

Oxidative decarboxylation of AKG by AKGD produces succinyl-CoA, which is further cleaved by succinyl-CoA synthase to generate succinate. Succinate can be further dehydrated by the enzyme succinate dehydrogenase (SDH), a four-subunit protein complex of SDHA, SDHB, SDHC and SDHD, to generate fumarate. Both succinate and fumarate are considered ‘oncometabolites’ along with 2HG, as they can also competitively inhibit AKG-dependent enzymes including the TET family of epigenetic regulating proteins [182]. Succinate levels increase under stresses such as hypoxia or hyperglycemia, or via dysfunction of SDH or fumarate overproduction [183,184,185]. Increased cellular succinate promotes tumor cell growth and survival through inhibition of HIF1-α prolyl hydroxylases resulting in HIF1α stabilization [186]. Succinate can be secreted [184] and is found in conditioned media from breast cancer cells [187]. This extracellular succinate acted in a paracrine manner to increase tumor cell invasion and polarize macrophages. Although not tested in breast cancer, exogenous succinate promoted metastatic tumor growth in vivo, which was partially dependent on HIF1α activity. Interestingly, tumor-associated macrophages downregulated SDH levels in tumor cells, thereby increasing succinate levels and HIF1α stabilization [188], highlighting reciprocal metabolic crosstalk within the tumor microenvironment. Pharmacological SDH inhibition using DT-010 impaired mitochondrial respiration, reduced ATP production and inhibited proliferation of breast cancer cells in vitro, and tumor growth in vivo [189].

Germline mutations in SDH subunits have been found to increase the risk of patients harboring PTEN mutations to develop cancer, including breast cancers [190], however SDH mutations are generally low in breast cancer [191,192]. Recently, SDHA expression was shown to be higher in breast cancer tumors compared to normal mammary tissue and associated with worse overall survival [193]. Consistent with this, SDHA levels are elevated in breast cancer cell lines compared to MCF10A cells, and its depletion decreased growth and colony formation in vitro [191,193]. In contrast to SDHA, SDHC levels are inversely correlated with EMT gene signatures and low SDHC levels trended to associate with worse overall survival in basal-like breast cancers [194]. Functional studies showed that heterozygous loss of SDHC resulted in increased EMT marker expression accompanied by impaired spheroid growth and migration in breast cancer cells. Given that SDH is comprised of four subunits encoded by distinct genes, assessing SDH complex activity may be a more reliable prognostic indicator. Although not assessed in breast cancer, serum succinate levels are higher in tumor bearing versus non-tumor bearing animals, and clinical studies found elevated serum succinate in lung cancer patients compared to healthy controls [187]. Although not yet assessed in breast cancer, these results suggest circulating succinate could be a useful prognostic marker. 

Fumarate accumulates in cells due to loss of fumarate hydratase (FH), the enzyme responsible for its conversion into malate [195], and fumarate accumulation results in its extracellular release [196,197], with implications for tumor progression and immune regulation. Tumor-derived extracellular fumarate suppressed CD8+ T-cell cytotoxicity by succinating the ZAP70 kinase to suppress T-cell function [198]. Increased intracellular fumarate also inhibits AKG-dependent enzymes including the TET family of epigenetic regulators, thereby contributing to tumor growth [182]. Fumarate can also participate in succination of proteins [199], and has been shown to modify PTEN with S-(2-Succinyl)cysteine (2SC), which disrupts its plasma membrane localization, preventing its ability to suppress PI3K/AKT signaling and thereby driving tumor cell growth [200]. 

Expression of FH is reported to be higher in breast cancer versus normal mammary tissue, and increased FH levels correlate with worse progression-free and overall survival [201]. No mutations in FH were found in familial breast cancer cohorts suggesting it is not a main driver of tumor initiation in this context [202]. In breast cancer cell lines, overexpression of FH promoted, while FH depletion inhibited cell proliferation and invasion in vitro, and impaired orthotopic tumor growth in vivo [201]. These results conflict with models suggesting a tumor-suppressor role for FH where its loss resulted in pro-survival metabolic adaptations and activation of oncogenic cascades mediated by increased fumarate levels [203]. Although a selective pharmacological FH inhibitor has been identified [204] and was shown to overcome the effects of exogenous fumarate on T-cell inhibition leading to restoration of effective anti-tumor T-cell killing of tumor cells in vitro [198], its direct effects on tumor cell growth in the absence of immune cells was not evaluated. Despite this, existing literature supports intrinsic and extrinsic roles for succinate and fumarate in directly modulating tumor cell growth and in modifying the activity of the tumor immune microenvironment.

#### 2.2.3. Malate and Malate Dehydrogenase 2

Malate is produced from the hydration of fumarate by the enzyme FH and is subsequently converted to oxaloacetate by malate dehydrogenases (MDH). Malate may have context-dependent effects in cancer, both inhibiting and promoting growth. There are two major MDH isoforms, MDH1 which is cytoplasmic and MDH2 which is mitochondrial [205]. Expression of MDH1 appears lower in breast cancer tumors compared to normal mammary tissue and shows no correlation with clinical outcome [206]. In contrast, MDH2 expression is elevated in breast tumors compared to normal mammary tissue and is upregulated in breast cancer cell lines compared to MCF10A cells [207]. MDH2 expression is the highest in TNBC, where high levels correlate with reduced immune cell infiltration, along with worse disease-specific and overall survival. Interestingly, an MDH2 variant (rs111879470) that affects the NAD-binding pocket of MDH2 has been linked to increased risk of breast cancer recurrence [208]. Increased expression of MDH2 in breast cancers may be partly explained by its upregulation in response to estrogen stimulation [209]. 

Supporting a role for MDH2 in breast cancer progression, depletion of MDH2 inhibited proliferation and colony formation while increasing apoptosis, whereas overexpression of MDH2 increased MDA-MB-231 cell growth in vitro and in vivo [207]. Interestingly, RNAseq and metabolomics data analysis suggested that one of the most significantly altered cellular pathways in MDH2 overexpressing cells was the PI3K/AKT/mTOR signaling pathway. Although not tested in breast cancer, LW6 and a derivative of this agent named compound 7 were found to inhibit MDH2 activity and growth of HCT116 colorectal cancer tumors in vivo [210,211]. These findings suggest that pharmacological inhibition of MDH2 may be a viable therapeutic option.

Although not an exhaustive description of the role of TCA cycle enzymes and metabolites in breast cancer, the evidence cited supports the importance of the TCA cycle and its metabolites in breast cancer progression by supporting bioenergetics, epigenetic modification and immunomodulation. Strategies to exploit metabolic vulnerabilities in this pathway by inhibiting activity of key enzymes or modulating metabolite levels could selectively impair tumor cell viability and energetic fitness while sparing normal tissue and should be pursued.

### 2.3. Pentose Phosphate Pathway and Nucleotide Synthesis

The pentose phosphate pathway (PPP) is a glycolysis-linked metabolic pathway that plays an essential role in cell growth and survival [212]. This pathway generates pentose phosphates for nucleotide synthesis and produces NADPH, which supports fatty acid synthesis and cell survival [213]. The PPP comprises two branches, the oxidative branch and the non-oxidative branch. In the oxidative branch glucose 6-phosphate (G6P) generated from glycolysis is irreversibly converted into 6-phosphogluconate by glucose-6-phosphate dehydrogenase (G6PD) [214]. The 6-phosphogluconate is further converted into ribulose-5-phosphate by 6-phosphogluconate dehydrogenase (6PGDH), generating CO_2_ and NADPH in the process. The non-oxidative branch produces glycolytic intermediates, including xylulose, that are converted into fructose 6-phosphate, glyceraldehyde 3-phosphate, and sedoheptulose-7-phosphate that reenter glycolysis, and ribose-5-phosphate which serves as a critical precursor for nucleic acid synthesis [215]. The PPP also produces NADPH which plays a vital role in maintaining cellular redox status [216,217]. Cancer cells can produce higher levels of ROS due to increased metabolic activity, however, rely on pathways that limit mitochondrial OXPHOS that produces a large amount of ROS by utilizing glycolysis [218] or the PPP which produces the ROS-detoxifying molecule NADPH [219]. As such, the PPP plays a crucial role in maintaining cellular redox homeostasis and providing nucleotide precursors to support rapid cellular proliferation.

#### 2.3.1. Glucose-6-Phosphate Dehydrogenase

G6PD the rate-limiting enzyme of the PPP is upregulated in breast tumors compared with normal mammary tissue and higher expression correlates with advanced stage and worse overall survival in patients [220,221,222]. G6PD expression is upregulated by mutant KRAS [223], MYC activity [224], PI3K/Akt signaling, loss of TP53 [9] or PTEN [225], or increased by HIF1α [226,227] and NRF2 [228] activity. Breast cancer cell lines also show increased G6PD levels compared to MCF10A cells [220,222,229], and G6PD depletion reduced cell viability, migration and colony formation in vitro [222,230], and orthotopic tumor growth and lung metastatic colonization in vivo [222]. As expected, depletion of G6PD resulted in increased levels of cellular ROS and decreased production of fatty acids contributing to decreased cell viability [230].

Pharmacologic G6PD inhibition using 6-aminonicotinamide (6AN) decreased cell viability and colony formation in vitro and tumor growth in vivo, which in one model was associated with reduced levels of tumor-infiltrating CD163+ M2-macrophages [220,222]. G6PD depletion or inhibition using the compound dehydroepiandrosterone (DHEA) or polydatin was also shown to confer sensitivity to doxorubicin or lapatinib in vitro [229,231]. Indeed, G6PD activity promotes STAT1-dependent cytokine secretion that polarized macrophages towards M2 phenotypes [220]. The plant-derived G6PD inhibitor polydatin [232], also increased cellular ROS and ER stress to induce autophagy in breast cancer cells, which was overcome by overexpression of G6PD [231]. Taken together, these findings support a central role for G6PD in breast cancer growth, redox regulation, immune modulation and response to therapy.

#### 2.3.2. 6-Phosphogluconate Dehydrogenase

6PGDH produces ribulose-5-phosphate to support nucleotide synthesis, making it an important contributor to tumor cell growth. 6PGDH levels are elevated in breast cancer cell lines compared to MCF10A, and upregulated in breast tumors compared to normal mammary tissue [233]. 6PGDH depletion reduced MCF7 cell proliferation and mammosphere formation in vitro, associated with decreased glucose consumption and increased glutamine consumption [234]. 6PGDH depletion also inhibited growth of MDA-MB-231 and MCF7 cells with little effect on MCF10A cell growth. Pharmacological 6PGDH inhibition using S3 [235], decreased cell viability and induced cell senescence [234], while 6PGDH inhibition using physcion, the parent compound of the S3 inhibitor [235], also sensitized tumor cells to paclitaxel or doxorubicin in vitro, and inhibited MDA-MB-231 tumor growth in vivo when administered alone or more effectively when used in combination with paclitaxel [233]. 

Clinically, 6PGDH is elevated in breast tumors compared to normal mammary tissue, with highest expression in Her2+ breast cancers [233,236]. Although one study did not observe association with patient survival [236], other reports suggest links between high 6PGDH levels and breast cancer recurrence [237,238]. While its association with patient prognosis remains unclear, 6PGDH levels are increased in epirubicin resistant compared to parental breast cancer cell lines [239], and 6PGDH depletion or inhibition with physcion resensitized epirubicin resistant cells to epirubicin via reduction in cellular NADPH levels. Taken together, available evidence suggests that 6PGDH promotes breast cancer growth and chemotherapy resistance supporting its therapeutic targeting in breast cancer.

In summary, the PPP is essential for breast cancer growth and progression through its ability to generate NADPH to support antioxidant defense and produce ribose-5-phosphate for nucleotide synthesis. Evidence supports that key enzymes, such as G6PD and 6PGDH, promote breast cancer cell proliferation, survival and drug resistance. Thus, PPP enzymes are emerging as promising prognostic biomarkers and therapeutic targets with the potential to enhance chemotherapy efficacy and overcome treatment resistance in breast cancer.

### 2.4. Fatty Acid and Lipid Production 

Although normal cells primarily use fatty acids (FA) obtained from the diet, cancer cells rely on de novo FA synthesis to meet their continual need for lipids to build cell membranes, support rapid cell division, and sustain signaling pathways [240,241]. Acetyl-CoA is required for de novo FA synthesis and can be generated through several metabolic pathways. Citrate from the TCA cycle can be cleaved by ATP-citrate lyase (ACLY) into acetyl-CoA and oxaloacetate [242]. Glutaminolysis of glutamine catalyzed by glutaminase (GLS) converts acetyl-CoA into glutamate which is subsequently converted to AKG by glutamate dehydrogenase (GDH), which then goes through a reverse flux in the TCA cycle to generate citrate [243]. In FA synthesis the generated acetylCoA is converted by ATP citrate lyase (ACL) and acetylCoA carboxylase (ACC) into malonylCoA, the substrate for fatty acid synthase (FASN) which ultimately generates palmitate, a foundational fatty acid used for membrane biogenesis, energy storage, and lipid-mediated signaling [244]. FAs are also catabolized by cells to generate energy through mitochondrial β-oxidation [245]. Although many cancers depend on FA metabolism, breast cancers are uniquely surrounded by FA-rich adipocytes, making them very reliant on FA metabolism for survival and progression. We will briefly highlight key enzymes regulating FA metabolism and their role in breast cancer progression. 

#### 2.4.1. Carnitine Palmitoyl Transferase 1A

Carnitine palmitoyl transferase 1A (CPT1A) is the rate-limiting enzyme enabling FA to enter mitochondria for β-oxidation and ATP production [246]. Located on the outer mitochondrial membrane, CPT1A transfers the acyl-group from acyl-CoA to carnitine to form acyl-carnitine. Acyl-carnitine can then be transported into the mitochondrial matrix by carnitine-acylcarnitine translocase (CACT), where it is subsequently processed back to acyl-CoA by carnitine palmitoyltransferase II (CPT2) and undergoes β-oxidation reactions.

Across public datasets, elevated CPT1A expression is associated with worse overall survival in breast cancer [247,248]. CPT1A is more highly expressed in ER+ vs basal breast tumors, and higher CPT1A at both the mRNA and protein levels is associated with higher proliferative capacity in ER+ tumors [247]. Interestingly, CPT1A is also secreted in tumor-derived exosomes [249], and CPT1A levels were higher breast cancer cell line conditioned media compared to that of transformed normal mammary epithelial cells [248]. Serum CPT1A levels are also higher in breast cancer patients relative to those with benign breast disease or healthy controls, and correlated with lymph node status, tumor size, TNM stage, histological grading, HER2 status, and molecular subtype [248]. Importantly, serum CPT1A levels decreased post-surgical tumor removal, suggesting its potential utility in monitoring disease. 

Given its central role in FA metabolism, depletion of CPT1A reduces mitochondrial respiration, ATP production, and FA β-oxidation [247]. CPT1A depletion impaired breast tumor cell colony growth and mammosphere formation [247,250], in part due to increased apoptosis, while its overexpression in non-tumorigenic MCF10A cells enhanced mammosphere formation [247]. CPT-family inhibitors etomoxir (CPT1A selective) or perhexiline (CPT1A and CPT2) dose-dependently inhibited tumor cell growth and survival in vitro, with etoxomir being particularly effective in inhibiting growth of tumors with high MYC activity (in MYC-expressing PDX tumor model and in the MYC-expressing transgenic MTB-TOM (MMTV-rtTA/TetO-MYC) model) in vivo [251]. In addition to inhibiting primary tumor growth, etoxomir also impaired metastatic colonization and tumor growth in orthotopic and metastasis seeding models [250]. Perhexiline also inhibited MDA-MB-468 tumor growth in vivo, in part via its ability to inhibit Her3 signaling [252]. As Her3 upregulation is associated with resistance to agents such as lapatinib, perhexiline administration in combination with lapatinib resulted in more durable inhibition of Her3 signaling. These findings suggest that targeting CPT1A may have significant therapeutic benefit in breast cancer, particularly those that appear to be most dependent on FA metabolism.

#### 2.4.2. Fatty Acid Synthase

FASN is a critical enzyme complex in de novo FA synthesis, which contains seven catalytic domains and generates palmitate from acetyl-CoA and malonyl-CoA in ATP and NADPH-dependent reactions [253]. Increased FASN was shown to correlate with disease-free and overall survival in breast cancer patients [254,255,256], however a large meta-analysis found no correlation between FASN and clinical outcomes, although it was associated with increased Her2 expression and larger tumor size [257]. Despite mixed prognostic results, FASN modulation consistently affects breast cancer growth. Depletion of FASN altered cellular metabolism, reducing glucose uptake and lactate production through downregulation of HK2 and PKM2 [254]. FASN depletion decreased cell viability and invasion in vitro [254,256,258], and although growth of primary orthotopic tumors was unaffected, metastases formation was significantly impaired [259]. FASN expression is also higher in metastatic compared to primary tumors in both preclinical models and patient samples, and its depletion inhibited brain metastases outgrowth [260]. Similarly, pharmacological FASN inhibition using α-mangostin, TVB-3166, TVB-2640, orlistat or diphenyleneiodonium (DPI) reduced cell viability in vitro [256,259,261,262,263], with orlistat and DPI effectively suppressing MDA-MB-231 xenograft tumor growth in vivo [256]. TVB-3166 also inhibited tumor growth of tamoxifen-resistant MCF7 tumors and restored tamoxifen sensitivity in vivo [262]. TVB-2640 also showed synergistic inhibition of cell and patient-derived organoid growth in combination with the topoisomerase inhibitor SN-38 [263]. Notably, in a Phase I clinical trial, breast cancer patients treated with TVB-2640 and paclitaxel achieved predominantly stable disease or partial responses [264].

Bioinformatic analyses of TCGA breast cancer datasets show FASN expression negatively correlates with infiltration of anti-cancer immune cells, cytolytic activity signatures and HLA-I expression [265]. Mechanistically, FASN supports PD-L1 stability by enabling its palmitoylation, and loss of FASN renders tumor cells more susceptible to T-cell-mediated killing [265]. Inhibition of FASN also enhanced the anti-tumor activity of a B7-H3 immune checkpoint inhibitor in syngeneic breast cancer models [266], suggesting that FASN promotes immune evasion in addition to supporting tumor metabolism. 

Because tumor cells preferentially rely heavily on de novo FA metabolism to support rapid proliferation, survival and therapy resistance, this pathway is an attractive therapeutic target. Key enzymes such as FASN and CPT1A show promise as both prognostic biomarkers and therapeutic targets and should be further investigated. 

### 2.5. Glutamine Metabolism

Glutamine metabolism is essential for energy production, biosynthesis, nitrogen transport, and redox homeostasis. Glutamine enters cells through glutamine transporters, including solute carriers (SLC) 1A5, SLC38A1, and SLC38A2 [267]. In the cytoplasm, glutamine serves as a precursor for asparagine, hexosamine, or nucleotide synthesis [243]. It can also be transported into mitochondria via an SLC1A5 variant, where it is converted to glutamate by GLS. Mitochondrial glutamate may be exported to the cytoplasm to support glutathione synthesis or further metabolized to AKG feeding into the TCA cycle to produce ATP. Because proliferating cancer cells rapidly deplete TCA intermediates, they rely on anaplerosis to replenish them. Glutamate is a key substrate for anaplerosis, with mitochondrial glutamate dehydrogenase 1 (GLUD1) converting glutamate into AKG, which releases ammonia that can modulate autophagy, buffer intracellular pH and serve as an alternative nitrogen source for amino acid synthesis [268]. The resulting AKG enters the TCA cycle, supporting sustained ATP production needed for rapid cell growth. 

Glutamine is the major nitrogen donor for de novo nucleotide synthesis, and its availability regulates pyrimidine and purine production [243,269]. In purine synthesis, two glutamine molecules are utilized to produce inosine monophosphate (IMP), the precursor of adenosine monophosphate (AMP), and guanosine monophosphate (GMP), with one additional glutamine molecule required to convert IMP to GMP. In pyrimidine biosynthesis, one glutamine molecule is consumed by the carbamoyl phosphate synthetase enzyme, and another is used to convert uridine triphosphate (UTP) into cytidine triphosphate (CTP). Recent studies show that cancer cells have increased shuttling of glutamine towards nucleotide biosynthesis to support the needs of rapid proliferation [269]. In breast cancer, glutamine dependence is most pronounced in TNBC cell lines which rely more heavily on glutamine metabolism than luminal subtypes [270]. Below, we will discuss the role of additional key metabolic enzymes in this pathway that influence breast cancer growth and progression.

#### 2.5.1. SLC1A5

SLC1A5 (aka ASCT2) is a neutral amino acid transporter which facilitates glutamine uptake. SLC1A5 expression is elevated in TNBC or Her2+ tumors compared to other subtypes [271,272,273], and is more highly expressed in tumors compared to normal breast tissue. Copy number gain or higher SLC1A5 expression also correlates with poorer cancer-specific and overall survival [273,274,275]. SLC1A5 is upregulated by MYC [276] and HIF2α, which promotes expression of a mitochondrial SLC1A5 variant [277]. Although SLC1A5 depletion led to reduced glutamine uptake in both TNBC and ER+ cells, cell growth was predominantly suppressed in TNBC cells, consistent with their stronger reliance on glutamine metabolism [271]. SLC1A5 depletion also impaired tumor engraftment and growth of HCC1806 xenografts. More recent studies have shown that growth of some luminal cell lines is also sensitive to SLC1A5 depletion or inhibition with gamma-l-glutamyl-p-nitroanilide (GPNA) [273]. SLC1A5 expression correlates with CD8+ T-cell, FOXP3+ Tregs and macrophage infiltration in breast tumors [274,278], and SLC1A5 depletion reduced PD-L1 expression [278], indicating that glutamine metabolism modulates immune evasion. Anti-estrogen therapies (such as tamoxifen and raloxifene) lower SLC1A5 levels and thus may exert some of their anti-tumor effect by suppressing glutamine metabolism [279]. High SLC1A5 expression predicts poor outcome in endocrine-treated patients, and SLC1A5 depletion sensitizes luminal breast cancer cells to tamoxifen [275]. Taken together, these findings identify SLC1A5 as a key regulator of glutamine metabolism, tumor growth, therapeutic response and immunosurveillance in breast cancer.

#### 2.5.2. Glutaminase and Glutathione

Tumor cells experience significant intrinsic and extrinsic stresses, including oxidative stress, nutrient deprivation, hypoxia, immune-mediated attack, and inflammation. Glutamine helps buffer these stresses by being converted through GLS into glutamate, which is then used by glutathione synthase (GS) to generate glutathione, a key antioxidant that detoxifies peroxides and maintains redox homeostasis [280]. Furthermore, glutathione can be recovered to its oxidized form through the conversion of NADP+ to NADPH. 

GLS catalyzes the rate-limiting step of glutaminolysis and is thus an important therapeutic target [268]. There are two isoforms of GLS, GLS1 and GLS2, whose expression is inversely correlated in breast cancer [281]. MYC upregulation in endocrine-resistant breast cancer increases glutamine metabolism via increasing SLC1A5 [282] and GLS1 expression [276], whereas wild-type p53 induces GLS2 expression [283]. GLS1 is most highly expressed in TNBC and basal-like tumors and is inversely correlated with ER and BRCA expression [281,284,285]. Immunohistochemistry further shows that GLS1 is elevated in invasive breast cancers compared to benign ductal carcinoma in situ (DCIS) and normal mammary tissue, while GLS2 is elevated in normal and DCIS tissues [286]. Additional studies also confirmed that GLS1 predominates in basal cancers, whereas GLS2 is more abundant in luminal subtypes [287]. Clinical associations remain unclear, as some studies report high GLS1 predicts better overall survival [281], whereas others link it to worse disease-free or overall survival [285,286,288]. In contrast, high GLS2 consistently associates with better disease-free survival [286]. These discrepancies may stem from differences in mRNA vs protein analysis, subtype bias, or failure to account for distinct GLS1 splice variants [284,289].

Despite the unclear correlation between GLS enzymes and clinical outcome, breast cancer cells heavily rely on glutamine metabolism for survival [290]. GLS1 depletion reduces cell viability and colony formation in vitro [291,292,293] and tumor growth in vivo [294,295]. Pharmacologic GLS1 inhibition using bis-2-(5-phenylacetamido-1,3,4-thiadiazol-2-yl)ethyl sulfide (BPTES) or CB-839 also impaired cell growth in vitro and xenograft or PDX tumor growth in vivo [284,295], and sensitized cells to carboplatin [285] and paclitaxel [296]. In syngeneic models, GLS1 depletion inhibited tumor growth in vivo by reducing glutamate levels and hence reducing its extracellular release which could otherwise impair T-cell functions [294,297]. GLS1 inhibitors appear to be most effective in non-luminal subtypes [298], whereas luminal breast cancers show greater sensitivity to GLS2 depletion or inhibition with a dual GLS1/GLS2 inhibitor 968 [298]. Supporting this, GLS2 overexpression promotes primary and metastatic tumor growth in vivo, and compensates for GLS1 loss, reducing TNBC sensitivity to GLS1 inhibitors [299]. Although a GLS1 inhibitor, IPN60090, entered Phase I clinical testing (NCT03894540), the trial was terminated, and results have not been reported. Taken together, these findings suggest that GLS plays a crucial role in modulating breast cancer growth and survival, but dual GLS inhibitors may have a broader clinical benefit in breast cancer. 

#### 2.5.3. Glutamate Dehydrogenase 1

GLUD1 (aka GDH), catalyzes the conversion of glutamate to AKG and plays an important role in glutamine anaplerosis. Although GLUD1 is often overexpressed and promotes EMT and drug resistance in other tumor types [300], its role in breast cancer is less well-defined. Higher GLUD1 is associated with lower grade ER+ tumors and only GLUD1 mRNA in ER+/Her2- tumors showed significant correlation with cancer-specific survival [301]. GLUD1 is also higher in invasive lobular breast cancers (ILC) compared to invasive ductal carcinomas [301,302], consistent with the slowergrowing, more indolent behavior of ILC. In contrast to these reports, GLUD1 levels are reported to be higher in breast tumor compared to normal mammary tissue, and correlated with increased stage and lymph node metastasis [272,303]. Functionally, GLUD1 inhibition using R126 increased ROS and decreased ER expression in tamoxifen-resistant ILC cells [302], although the effects on cell viability were not detailed. GLUD1 depletion or inhibition with R126 also significantly inhibited MDA-MB-231 cell growth and colony formation in vitro, likely due to impaired redox homeostasis [303]. Additionally, GLUD1/2 depletion reduced levels of downstream amino acids (such as proline and aspartate) which may further impair tumor cell growth [304]. GLUD also plays a role in recycling ammonia derived from glutaminolysis to support cell survival under high ammonia conditions. In fact, exogenous ammonia promoted tumor cell growth and colony formation, whereas GLUD depletion mitigated these effects leading to reduced tumor growth in vivo [304]. Although the role of GLUD as a prognostic marker in breast cancer remains unclear, evidence suggests inhibition of GLUD1 may remain an important therapeutic strategy in breast cancer.

In summary, glutamine metabolism is essential for breast cancer progression by supplying key metabolic intermediates such as nucleic acids and amino acids needed for rapid proliferation, and by maintaining redox homeostasis via glutathione production. Given that many breast cancers appear to have ‘glutamine addiction’, continued evaluation of therapeutic strategies targeting this pathway is warranted. 

### 2.6. Serine Metabolism

Increased serine biosynthesis is a common alteration in cancer cells [305]. Serine supports the biosynthesis of multiple biomolecules, such as glycine and cysteine. Glycine is a precursor of porphyrins, purine nucleotide bases and glutathione. During the conversion of serine to glycine, serine hydroxymethyltransferase (SHMT) donates a one-carbon unit to tetrahydrofolate, producing 5,10-methylenetetrahydrofolate, a key folate intermediate required for purine synthesis. Thus, increased serine availability can fuel rapid cancer cell proliferation. Cells acquire serine either through uptake from the extracellular environment or through de novo synthesis from glucose. Three main enzymes catalyze reactions in the serine synthesis pathway: phosphoglycerate dehydrogenase (PHGDH), phosphoserine aminotransferase (PSAT), and phosphoserine phosphatase (PSPH). A proportion of 3-phosphoglycerate generated from glycolysis is converted to 3-phosphohydroxypyruvate (3PHP) by PHGDH [306]. PSAT then converts 3PHP into 3-phospho-serine, which is dephosphorylated by PSPH into serine. Below, we will highlight how key enzymes in this pathway promote breast cancer progression.

#### 2.6.1. Serine Hydroxymethyltransferase

SHMT catalytically converts serine to glycine to generate NADPH and 1-carbon metabolites required for nucleotide synthesis. There are two SHMT isoforms, SHMT1 which localizes to the cytoplasm, and SHMT2 which functions in the mitochondria, and is the predominant isoform supporting one-carbon metabolism in rapidly proliferating cells [307]. A recent meta-analysis showed that elevated SHMT2 levels are associated with worse outcomes across multiple cancers including breast cancer [308]. SHMT2 expression was predominantly elevated in TNBC, HER2+ and luminal B breast cancers compared to luminal A breast cancers and normal mammary tissues, and high SHMT2 correlates with advanced stage and poorer progression-free and overall survival [309,310,311,312]. In contrast, higher SHMT1 expression correlated with better disease-free survival [311]. SHMT expression is upregulated by MYC [313], HER2 [314], estrogen-related receptor α [315] and mutant p53 variants such as R280K [16]. SHMT2 is also elevated in lapatinib-resistant breast cancer cells [315].

In preclinical models, metastatic subclones of MDA-MB-231 cells displayed reduced SHMT1 but elevated SHMT2 expression compared to parental cells [311]. SHMT2 depletion significantly impaired growth of these metastatic subclones in vitro, and inhibited orthotopic or lung metastatic tumor growth in vivo. Conversely, SHMT2 overexpression promoted breast cancer cell line growth and colony formation in vitro and tumor growth in vivo, while its depletion inhibited it [312]. SHMT2 also regulated VEGF expression, suggesting part of its in vivo tumor-promoting ability may be mediated through increased angiogenesis. SHMT2 depletion also sensitized breast cancer cells to lapatinib [315]. The antidepressant drug sertraline was identified as a SHMT inhibitor, and dose-dependently inhibited serine metabolism-dependent breast cancer cell growth in vitro and in vivo, with greater efficacy when combined with the mitochondrial inhibitors rotenone, antimycin A or artemether [316]. Additional SHMT2 inhibitors (e.g. SHIN1/2, or pyrrolo[3,2-*d*]pyrimidine) suppressed growth and sensitized tumors to methotrexate [317], 5-fluorouracil [318] or cisplatin [319] in other tumor types, however, these combinations have not yet been tested in breast cancer. Together, current evidence suggests elevated SHMT2 promotes breast cancer progression, while SHMT2 inhibition impairs tumor growth and enhances sensitivity to anti-cancer agents. However, breast tumor cells with greater dependence on serine metabolism are likely to be the most responsive to SHMT2-directed therapies.

#### 2.6.2. Phosphoglycerate Dehydrogenase

PHGDH is upregulated in several cancers and is often associated with tumor aggressiveness [320]. The PHGDH gene is located on chromosome 1p, a region frequently amplified in breast cancer, and PHGDH is most highly expressed in TNBC and ER-negative breast cancers [321,322]. Elevated PHGDH has been linked to worse disease-free survival in some studies [321], although others report that low or heterogenous PHGDH expression correlates with shorter disease-free, metastasis-free or overall survival [323,324]. PHGDH expression is upregulated by MYC [325] and ATF4/ATF3 [326] and suppressed by p53 [327]. Post-transcriptional regulation of PHGDH also occurs, as epithelial splicing regulatory protein 1 (ESRP1) stabilizes PHGDH mRNA by binding to its 5’ untranslated region [328], while the lncRNA PlncRNA-1 reduces PHGDH protein levels [329].

Ectopic expression of PHGDH in non-tumorigenic MCF10A cells disrupted apical polarity, produced lumen-deficient mammospheres, and enabled matrix-attachment independent survival, features suggestive of malignant transformation [330]. However, PHGDH expression appears dynamic, with lower levels in circulating tumor cells (CTC), and in early metastatic lesions than in primary mammary tumors or established lung metastases in preclinical breast cancer models [323]. Consistent with this, patient lymph node metastases had lower PHGDH expression compared to matched primary tumors. Functionally, depletion of PHGDH increased cell invasion and lung metastatic burden in a syngeneic breast cancer model [323]. Other studies have reported that PHGDH depletion inhibited cell growth and colony formation in vitro [328,331], but had no effect on primary tumor growth in vivo [331], or its depletion or pharmacological inhibition using NCT-503 or CBR-5884 impaired tumor cell growth in vitro and xenograft tumor growth in vivo, but only in tumors dependent on glucose-derived serine [332,333,334]. Depletion of PHGDH was also shown to sensitize TNBC cell lines to doxorubicin in vitro and in vivo [335]. Overall, while the role of PHGDH may differ between proliferative or metastatic contexts, serine metabolism remains an attractive therapeutic target. Given serine is a critical precursor for nucleotide synthesis, one-carbon metabolism, and antioxidant defense, PHGDH inhibition may be effective in tumors relying on serine biosynthesis or when used in combination with other anti-cancer agents.

## 3. Conclusions and Future Directions

Targeting breast cancer metabolism remains a promising therapeutic strategy, as metabolic rewiring is a hallmark of tumor progression and treatment resistance. Key pathway targets and their role in breast cancer are summarized in Table 1. Inhibiting key pathways such as glycolysis, glutaminolysis, one-carbon metabolism, nucleotide synthesis and fatty acid metabolism, may disrupt the bioenergetic and biosynthetic demands of rapidly proliferating cancer cells and enhance effectiveness of existing therapies. However, several challenges remain. Metabolic plasticity enables cancer cells to compensate via alternative pathways, and systemic inhibition risks toxicity in metabolically active normal tissues. Furthermore, metabolic dependencies vary across tumor subtypes and microenvironmental contexts, necessitating biomarker-driven patient selection and combination strategies to maximize therapeutic benefit while minimizing adverse effects. Despite these challenges, exploiting metabolic vulnerabilities in breast cancer remains a promising avenue to improve treatment response and long-term breast cancer outcomes.

## Figures and Tables

**Table 1:** Summary of metabolic alterations and vulnerabilities in breast cancer.

Metabolic Pathway	Enzyme	Alteration in Breast Cancer	Subtype Associations	Therapeutic Vulnerability
**Glycolysis**	**HK2**	Upregulated [36,37,41,42] Required for tumor initiation and maintenance [52] Contributes to immune evasion [68]	Upregulated in HER2+ breast cancers [38]	Depletion of HK2 increases tumor cell apoptosis [74] and increases sensitivity to radiotherapy and chemotherapy [53,72,75], and oncolytic virus therapy [73]
	**PFK**	PFKP upregulated and associated with worse prognosis [85,86,87,88]	Enriched in TNBC [87,88]	Impairing PFKP activity decreased cell invasion and tumor growth in vitro and in vivo [87,92,93,94]
		PFKFB3 overexpressed and associated with worse prognosis, increased metastasis and resistance to chemotherapy [100,106,107,108,109]	Upregulated in TNBC and HER2+ breast cancers [100,106,107]	PFKFB3 inhibition impairs tumor growth in vitro and in vivo [100,110] and sensitizes tumor cells to chemotherapy [111]
		PFKFB4 and associated with worse prognosis [113,114,115,116] Increased expression associated with resistance to Palbociclib [121]	Highly upregulated in TNBC [115]	PFKFB4 inhibition reduces breast cancer tumor growth in vivo [113,120,122]
	**LDH**	LDH-A/C elevated [131,132,157] LDH-B decreased [155] Increased LDH-A associated with worse prognosis and tamoxifen resistance [131,132,133,134,141] LDH-C contributes to immune evasion [158] Total circulating LDH levels associated with worse prognosis [127,128,129,130]	LDH-C predominantly elevated in TNBC [157]	Inhibition of LDH-A or C inhibits in vitro and in vivo tumor growth and metastasis [131,139,140,142,144,145,146,147,148,157] and can sensitize to anti-cancer drugs [159]
**TCA Cycle**	**AKG & IDH**	AKG inhibits tumor cell growth [167,168] IDH2 elevated and associated with worse prognosis [175,176,178] Mutations in IDH increase 2HG production from AKG to promote tumor growth [170,171]	IDH2 elevated in TNBC [178] Although rare IDH1 mutations have been found in ER+ and SPCRP breast cancers [173,174]	Depleting IDH2 inhibited tumor growth and sensitized tumor cells to doxorubicin [178]
	**SDH & FH**	SDHA elevated and linked to worse prognosis [193] SDHC inversely correlated with EMT [194] FH increased and associated with worse prognosis [201]	Variable across subtypes	Targeting SDH/FH-related pathways inhibits tumor growth in vitro and in vivo [189,201]
	**MDH1/2**	MDH2 overexpressed and associated with worse prognosis [207] MDH1 decreased [206]	High MDH2 in TNBC [207]	MDH2 inhibition suppresses tumor growth [207]
**Pentose** **Phosphate Pathway**	**G6PD**	Upregulated and associated with worse prognosis [220,221,222]	Elevated in most subtypes	Inhibition induces ROS-mediated cell death, decreased cell migration and viability [220,222,230] Inhibition sensitizes tumors to chemotherapy [229,231]
	**6PGDH**	Elevated expression [233] Confers chemotherapy resistance [239]	Highly expressed in HER2+ tumors [236]	Inhibition reduces tumor cell growth in vitro and in vivo and sensitizes tumors to chemotherapies [233,234,235]
**Lipid** **Metabolism**	**CPT1A**	Increased expression and associated with worse prognosis [247,248]	Enriched in ER+ breast cancers [247]	Depletion impairs tumor growth and metastatic colonization [247,250,251,252]
	**FASN**	Upregulated but discrepant results for its association with prognosis [254,255,256,257]	High in HER2+ breast cancers [257]	Inhibition reduced tumor growth and metastasis and sensitized to tamoxifen [254,256,258,259,261,262,263] Pharmacological inhibition led to stable disease or partial responses in breast cancer patients [264]
**Glutamine** **Metabolism**	**SLC1A5**	Amplified expression [273,274,275] Associated with immune infiltration [274,278]	High in TNBC and HER2+ [271,272,273]	Depletion reduces tumor growth and sensitizes cells to tamoxifen [271,273,275]
	**GSH & GS**	Elevated with majority of studies suggesting high GLS1 associated with worse prognosis [285,286,288] Increased GLS2 expression associated with better prognosis [286]	GLS1 enriched in TNBC [281,284,285] GLS2 enriched in luminal [287]	Disrupting glutathione metabolism sensitizes tumors to oxidative stress and inhibits tumor growth in vitro and in vivo [284,285,291,292,293,294,295,296,298]
	**GLUD1**	Conflicting results suggesting high expression associated with lower grade breast cancer [301,302]; with other studies suggesting it is increased in breast cancer and associated with lymph node metastasis [272,303]	Correlated with ER+ [301]	GLUD1/2 depletion results in increased cellular ROS and reduced tumor cell growth [303,304]
**Serine** **Metabolism**	**SHMT**	Increased SHMT2 expression associated with worse prognosis [308,309,310,311,312] Increased SHMT1 expression associated with better prognosis [311]	Elevated in HER2+, TNBC, and luminal B breast cancers [308]	Targeting SHMT impairs proliferation and tumor growth and sensitizes to chemotherapy [311,312,315,316]
	**PHDGH**	Amplified [320] Conflicting results suggesting associated with worse [321] or with better prognosis [323,324]	Enriched in TNBC and ER-negative breast cancers [321,322]	Inhibition reduces tumor growth and sensitizes cells to chemotherapy [328,331,332,333,334,335]

## Data Availability

No new data were created or analyzed in this study.
